# Radiochemistry: A Hot Field with Opportunities for Cool Chemistry

**DOI:** 10.1021/acscentsci.3c01050

**Published:** 2023-11-14

**Authors:** Gregory D. Bowden, Peter J. H. Scott, Eszter Boros

**Affiliations:** † Department of Radiology, University of Michigan, 1301 Catherine, Ann Arbor, Michigan 48109, United States; ‡ Werner Siemens Imaging Center, Department of Preclinical Imaging and Radiopharmacy, Eberhard Karls University Tuebingen, 72074 Tuebingen, Germany; § Cluster of Excellence iFIT (EXC 2180) “Image Guided and Functionally Instructed Tumor Therapies”, Eberhard Karls University of Tuebingen, 72074 Tuebingen, Germany; ∥ Department of Chemistry, University of Wisconsin, 1101 University Avenue, Madison, Wisconsin 53706, United States

## Abstract

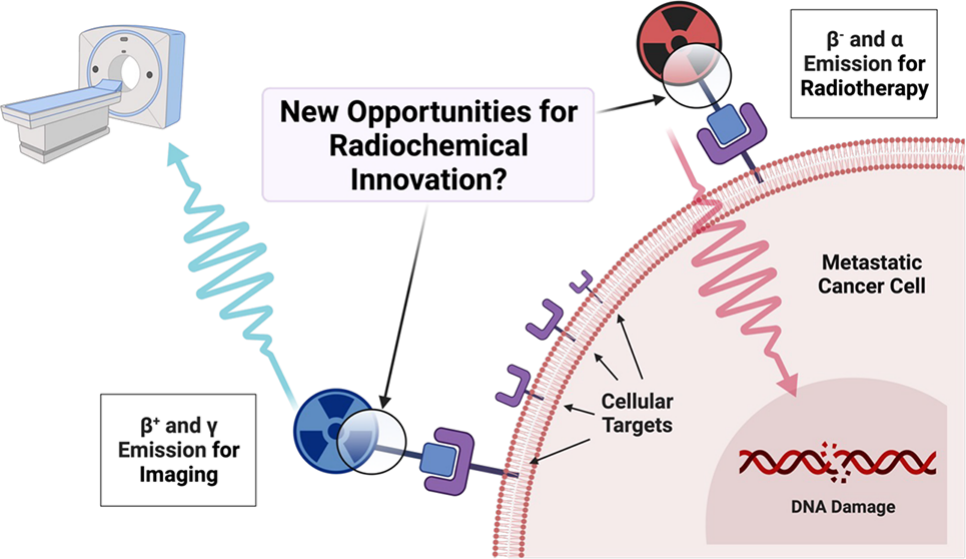

Recent Food and Drug Administration (FDA) approval of diagnostic and therapeutic radiopharmaceuticals and concurrent miniaturization of particle accelerators leading to improved access has fueled interest in the development of chemical transformations suitable for short-lived radioactive isotopes on the tracer scale. This recent renaissance of radiochemistry is paired with new opportunities to study fundamental chemical behavior and reactivity of elements to improve their production, separation, and incorporation into bioactive molecules to generate new radiopharmaceuticals. This outlook outlines pertinent challenges in the field of radiochemistry and indicates areas of opportunity for chemical discovery and development, including those of clinically established (C-11, F-18) and experimental radionuclides in preclinical development across the periodic table.

## Abstract 2

This outlook discusses how the demand for theranostic radiopharmaceuticals will require new chemical discoveries to couple therapeutic and imaging radionuclides to pharmaceutically relevant molecules.

## 1 Introduction

Nuclear medicine employs radiopharmaceuticals, bioactive molecules labeled with a radionuclide, for diagnostic imaging and radiotherapy. The choice of radionuclide depends on (a) the molecule to be labeled and (b) the intended application. When deciding on an appropriate radionuclide with which to label a given molecule, a general rule of thumb is to match the physical half-life of the radionuclide with the biological half-life of the molecule. Thus, short-lived radionuclides (*t*_1/2_ = minutes to hours) are appropriate for small molecules with fast kinetics, while longer-lived nuclides (*t*_1/2_ = hours to days) are more applicable for larger molecules and biologics, such as antibodies and their various fragments, with slower circulation times. Moreover, for diagnostic imaging, radiopharmaceuticals need to be labeled with β^+^-emitting radionuclides for positron emission tomography (PET) imaging, while γ-emitters are selected for single-photon emission computed tomography (SPECT). Radiopharmaceuticals for therapy are labeled with α, β^–^ and Auger-emitters. Our institutions have been employing radiopharmaceuticals for imaging and therapy since the 1940s, including the introduction of ^131^I-meta-iodobenzylguanidine (MIBG) and ^131^I-tositumomab (Bexxar) in the 1980s and 1990s.^1,2^

**Figure 1:**
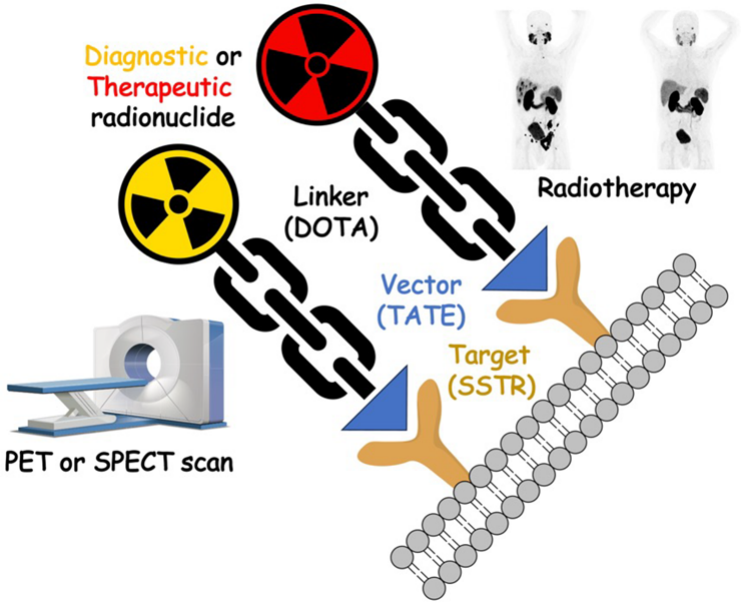
Schematic description of the theranostic concept with radionuclides.

During the 2000s, there was a lull in the translation and regulatory approval of new radiopharmaceuticals, in part because of the changing regulatory environment for such drugs in the United States at that time due to the FDA Modernization Act. Nevertheless, establishing dedicated regulatory pathways opened the door for the commercial development of radiopharmaceuticals, and there are now dozens of FDA-approved radiopharmaceuticals for diagnostic imaging and therapy (Figure 1).^3,4^ At the same time, the concept of theranostics has emerged.^5^ A theranostic agent (or pair, Figure 1) is a combination of a therapeutic and a diagnostic. Theranostics consist of a pair of (in some cases, chemically identical) radiopharmaceuticals with the same targeting vector—one labeled with a diagnostic radionuclide and the other a therapeutic. The diagnostic agent is used initially to confirm target expression and eligibility for treatment. Patients then receive several cycles of the radiotherapeutic over several months (e.g., 4–6 cycles every 6 weeks), with additional diagnostic scans throughout treatment and/or following the final dose to confirm response as necessary.

Pairs of theranostics have been approved by FDA for neuroendocrine tumors (targeting the somatostatin receptor 2, SSTR2) and prostate cancer (targeting the prostate-specific membrane antigen, PSMA), and early signs point to transformative care for patients with both types of cancer, which is resulting in unprecedented demand for the new agents.^6,7^ This new age of theranostics has transformed nuclear medicine, essentially overnight, into a multibillion dollar industry, creating an investment windfall for the field, taking a historically academic discipline into the realm of Big Pharma and ushering in a race to develop new theranostic agents for many other types of cancer.^8−10^

**Figure 2:**
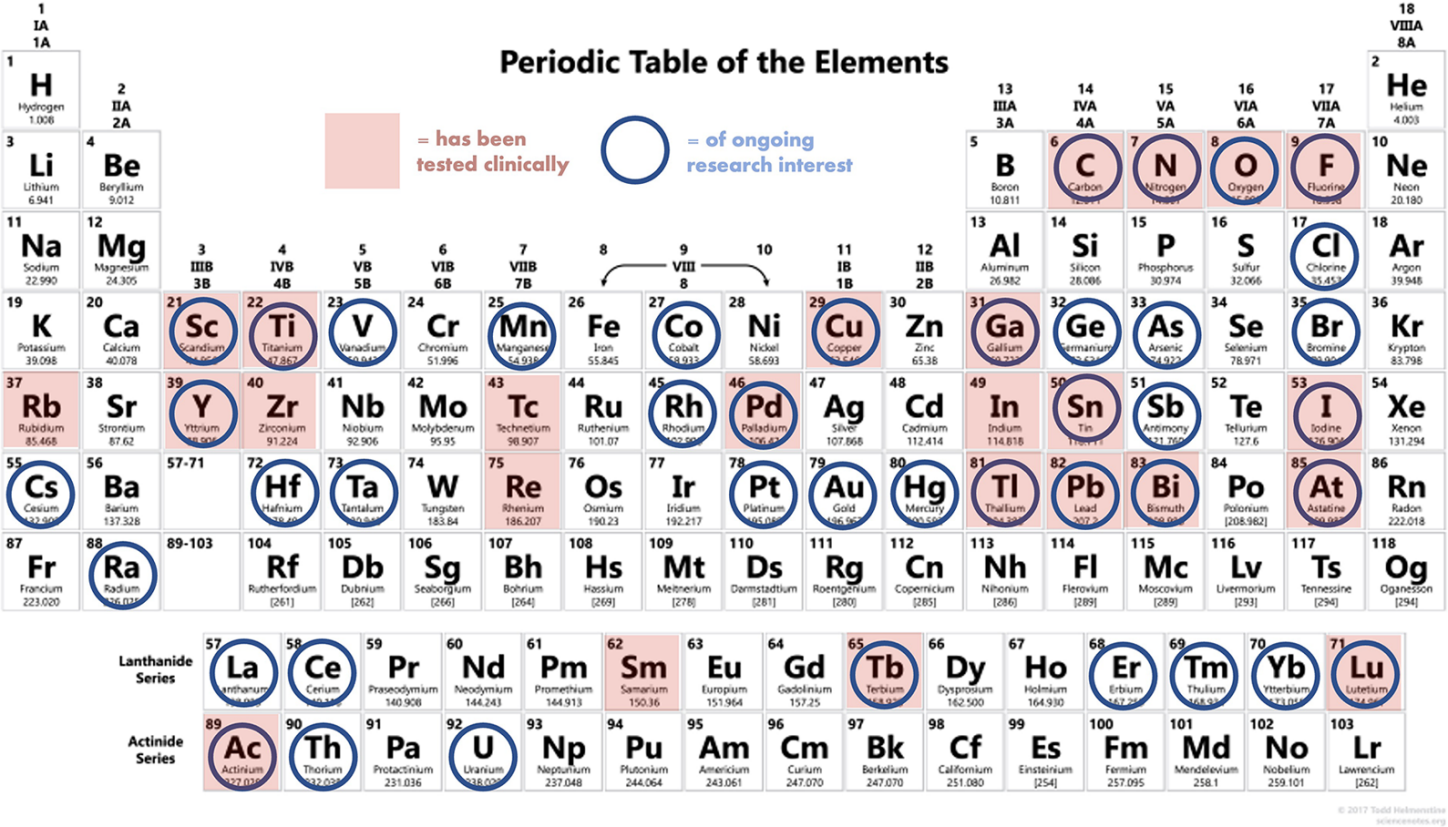
Summary of elements which possess radioisotopes of clinical and preclinical research interest for nuclear medicine, sometimes referred to as the “nuclear chocolate box of elements,” a phrase coined in the review by P. J. Blower.^14^

Critical to fully unleashing the power of theranostics against cancer are the disciplines of radionuclide production and radiochemistry. Both fields are enjoying somewhat of a renaissance, in part due to this rapid growth in theranostics, but also the increasing use of PET/SPECT imaging in drug discovery and personalized medicine.^11−13^ Radionuclide production is going to be critical in the coming years to ensure there is an adequate supply of both diagnostic and therapeutic radionuclides, while radiochemistry is essential for enabling the incorporation of said radionuclides into an increasingly diverse chemical space, including traditional small-molecule pharmaceuticals, larger biologics, and nanoplatforms. There also remain questions about the right choice of theranostic pairs, including the need to label both small molecules (e.g., ^18^F/^11^C/^211^At, ^76^Br/^77^Br, ^123^I/^124^I/^131^I) and large peptides/biologics (^68^Ga/^177^Lu/^225^Ac), as well as the need for “true” (or “matched”) theranostic pairs that consist of isotopes of the same element (e.g., ^61,64^Cu/^67^Cu, ^43^Sc/^47^Sc, ^86^Y/^90^Y, ^203^Pb/^212^Pb). Development of any (or all) of these radionuclides for theranostic applications involves a complex interplay between the isotope supply, availability of appropriate radiochemistry labeling methods and/or chelation chemistry, intellectual property landscape, and regulatory pathway considerations (Figure 2).^14^ In this Outlook, we consider the current landscape for theranostics as it pertains to these considerations and where, as chemists, we still need to invest R&D efforts to ensure the success of the field for cancer (and other) patients the world over.

## 2 Main Group Elements for Imaging and Therapy

### 2.1 Production of PET Tracers by Late-Stage Labeling with ^11^C, ^13^N, and ^18^F

Carbon-11 and fluorine-18, and to a lesser extent, nitrogen-13, are well-established radionuclides with excellent imaging properties for both clinical PET imaging and preclinical research.^15^ Both radionuclides are routinely produced globally by a growing network of medical cyclotrons and radiopharmacies for the synthesis of a variety of PET radiotracers and radioligands. In particular, ^18^F, in the form of [^18^F]fluorodeoxyglucose ([^18^F]FDG), a radiotracer for the abnormal glucose metabolism characteristic of most cancerous tissues, remains the workhorse of routine clinical PET imaging.^16,17^ The success of [^18^F]FDG-PET imaging has driven the development of numerous other innovative radiopharmaceuticals for clinical diagnosis and preclinical research and has cemented nuclear medicine’s place in mainstream clinical practice.

As short-lived radioisotopes of the ubiquitous main group elements carbon, nitrogen and fluorine, ^11^C (*β*^*+*^, *t*_1/2_ = 20 min), ^13^N (*β*^*+*^, *t*_1/2_ = 10 min), and ^18^F (*β*^*+*^, *t*_1/2_ = 110 min) are ideal for the high molar activity radiolabeling of small molecules with short *in vivo* half-lives. Moreover, recent years have seen an expansion in the number of radiochemical methodologies for the late-stage incorporation of these radionuclides into useful synthons and complex pharmaceutically relevant molecules.^18−21^ New methodologies, such as the popular copper-mediated radiofluorination reaction, continue to expand the chemical space available to radiochemists and imaging scientists, which has, in turn, resulted in an expanded target space, presenting imaging scientists, clinicians, and radiochemists with new opportunities for the imaging of novel targets via novel imaging approaches.^22,23^ Additionally, new methodologies afford radiochemists more freedom and flexibility to synthesize biologically relevant molecules without compromising on structure or performance. As the field moves into the theranostic era, the unparalleled potential for chemical diversity afforded by ^11^C-, ^13^N-, and ^18^F-based radiopharmaceuticals will be critical to rapidly identify and leverage new theranostic targets, particularly those residing across the blood-brain barrier.

However, while the growing list of new ^11^C, ^13^N, and ^18^F radiochemical methodologies continues to provide unprecedented access to new radiopharmaceutical diversity, the most efficient way to navigate that radiochemical space, both in terms of how we choose which molecules to study and how we choose to label them for meaningful GMP radiotracer production, is still an open question.^24^ Data-driven tools, including statistical experimental design (Design of Experiments, DoE), artificial intelligence (AI), and machine learning (ML), are expected to play a crucial role in every stage of the tracer development pipeline (Figure 2).^25−27^ This includes identifying lead candidate molecules and targets, pinpointing metabolically appropriate labeling sights, as well as planning and mitigating risks and failure points in radiosynthetic pathways for GMP production. These tools will help accelerate novel radiopharmaceutical concepts through development and into production for preclinical and clinical use. How data science, AI, and ML can be best leveraged to explore, study, and optimize new chemistry for reaction discovery and drug development is an ongoing area of research in the broader chemistry and medicinal chemistry fields. While automated workflows and smart laboratories interconnected through the Internet are becoming more commonplace, the acquisition, curation, and utilization of large and reliable chemical data sets remain a significant challenge.^28^

The field of radiochemistry is, however, uniquely situated to overcome these challenges and fully leverage chemical data science to develop new theranostic radiopharmaceuticals. Radiochemists typically deal with a smaller subsection of broader chemical space, limited by the chemical and physical constraints imposed using short-lived radionuclides like ^11^C, ^13^N, and ^18^F. Additionally, the radiopharmaceutical community is relatively small and well-connected, making the exchange of reliable chemical data more feasible. To this end, several ongoing research efforts are underway to develop new radiochemical techniques to rapidly and efficiently explore and map radiochemical space.^29,30^ Moreover, the lessons learned while applying and leveraging the data science revolution for the discovery and development of novel theranostic radiopharmaceuticals have the potential to impact the broader pharmaceutical field.

### 2.2 Heavy Radiohalogens

The tracer discovery efforts and radiochemical advances made using ^11^C, ^13^N, and ^18^F chemistry will inform how we choose and apply other theranostic isotopes. In addition to fluorine-18, the heavy radiohalogens, namely, bromine, iodine, and astatine, have played, and will continue to play, an important role in the ever-developing theranostic landscape.^31^

### 2.3 Radioiodine: The Old Guard of Nuclear Medicine

Iodine-131 (EC, *β*^*–*^, *t*_1/2_ = 8.02 days) iodide was the first readily available radionuclide widely used as a radiotherapy for thyrotoxicosis and thyroid cancer.^32^ With the invention of the gamma camera in 1957, ^131^I uptake could be directly imaged, heralding it as the first true theranostic agent.^33,34^ Since then, radiopharmaceuticals labeled with other isotopes of iodine, namely ^124^I (*EC*, *β*^*+*^, *t*_1/2_ = 4.18 days) and ^123^I (*EC*, *t*_1/2_ = 13.2 days), have been applied for both PET and SPECT imaging, respectively. ^125^I (EC, *Auger e*-, *t*_1/2_ = 59.4 days) is commonly used as an Auger emitter for brachytherapy and as a radiolabel for preclinical radiological assays.^31^ Due to their relatively long half-lives, radioiodinated pharmaceuticals can be produced and shipped to clinics not equipped for radiopharmaceutical production.^35^ The application of radioiodine for theranostic applications also benefits from a well-established and expanding toolbox for the iodination of larger proteins and small molecules.^36^ However, despite their long history in clinical nuclear medicine, many common iodinated radiopharmaceuticals suffer significant drawbacks with regard to complex production (volatile radioiodine), *in vivo* stability issues, complex and undesirable decay pathways and energies, or biologically incompatible half-lives.^33,37^ This has spurred research into alternative radiohalogen theranostic pairs.

### 2.4 ^76/77^Br: Has Their Time Come?

Positron emitting bromine-76 (*β*^*+*^, *t*_1/2_ = 16.2 h) and the auger electron emitter bromine-77 (*Auger e*^*–*^, *t*_1/2_ = 57 h) are well-suited as a “true theranostic pair” for the isotopic radiolabeling of small molecules against intracellular targets. The diagnostic and therapeutic versions of the same radiopharmaceutical are chemically indistinguishable and thus have identical pharmacological properties.^38^ This provides a significant advantage when designing, producing, evaluating, and approving novel theranostic compounds for clinical use. While the development of labeling methodologies specific to radiobromine has not received the attention afforded to the other radiohalogens, bromine chemistry is generally versatile and well-understood. Thus, efforts are underway to adapt these chemistries to the production of novel radiobrominated radiopharmaceuticals.^39^ The bromide anion is significantly more nucleophilic than fluoride for use in nucleophilic substitution reactions, while, like iodine, it can be oxidized to form high molar activity electrophilic bromine sources.^31^ Organobromides are also typically more stable than their organoiodide counterparts, thus demonstrating generally better overall in vivo stability.^40^

**Scheme 1:**
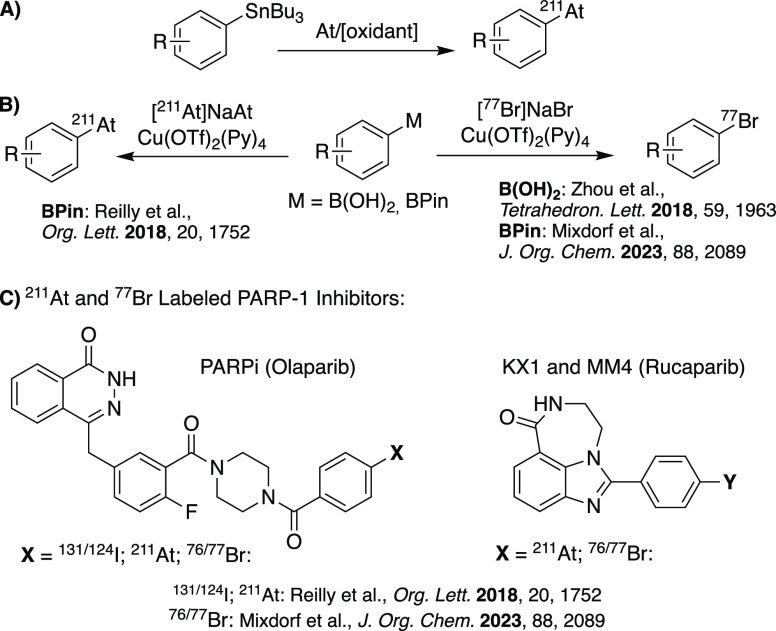
(A) Astatination via the Decannulation of Organotin Precursors, (B) Recent Metal-Mediated Approaches for Radiobromination and Astatination, (C) Poly(ADP-ribose) Polymerase (PARP) Is an Attractive Intracellular Target for At and Br Bearing Small-Molecule Radiopharmaceuticals Based on the PARP Inhibitors Olaparib and Rucaparib

The predominant factor limiting the clinical application of ^76^Br and ^77^Br is their production. Both radionuclides are typically produced from selenium targets via the ^76/77^Se(p,n)^76/77^Br nuclear reactions; however, the target physics and isotope isolation procedures (via thermal chromatographic distillation) pose significant challenges that limit production capacity. Selenium has poor electrical and thermal conductivity and high volatility, making it intolerant to even low-intensity proton bombardment.^38^ As such, it must be stabilized as an intermetallic alloy using other metals such as copper, nickel, zinc, or cobalt.^40^ The use of these metals results in the coproduction of unwanted radioactive byproducts, such as zinc-63 and copper-60, that must be separated from the product bromide for recycling or disposal. The number of sites capable of producing ^76^Br and ^77^Br is, therefore, currently limited; however, both isotopes have sufficiently long half-lives that allow them to be shipped moderate distances from centralized production sites. Higher-yielding production strategies have been proposed using heavy ion bombardment techniques, but the number of cyclotrons capable of performing these nuclear reactions is even more limited.^38^ Therefore, the development of new cyclotron targetry techniques and radionuclide isolation protocols to produce ^76^Br and ^77^Br are being actively investigated to meet the increasing preclinical and clinical demand.^40^ Additionally, interest in radiobromine labeling chemistry has seen a resurgence, and several groups are working on adopting modern metal radiohalogenation methods for use with radiobromine (Scheme 1).^39,41,42^

### 2.5 ^211^At: Chemistry of Elements without Stable Isotopes

Astatine-211 (α, *t*_1/2_ = 7.21 h) has been earmarked as a highly promising radionuclide for targeted alpha therapy, with F-18 proposed as a potential diagnostic partner.^43^^211^At can be synthetically produced via the α-beam irradiation of bismuth-209 with higher energy cyclotrons (>25 MeV).^44^ While ^211^At can be produced in relatively good yields, the limited number of cyclotrons capable of producing the radionuclide will make distribution for (pre)clinical trials a significant challenge.^45^ Additionally, astatine is the rarest naturally occurring element on Earth, present only as very short-lived daughter radionuclides from heavier element decay or as short-lived synthetic isotopes. The longest-lived isotope of astatine, ^210^At, has a half-life of only 8.1 h. Therefore, the chemistry of astatine has been studied only in low-concentration radiochemical experiments and is not fully understood.^46^ At^–^ exhibits chemistry akin to iodide; however, it is also easily oxidized to At^(I)^, which exhibits metalloid-like behavior.^47^ At^(III)^ has also been observed but was found to be difficult to isolate from At^(I)^ species. At^(V)^ has been demonstrated under highly oxidizing conditions but has not yet been shown with functional complexation chemistry. The At^(VII)^ oxidation state has been theorized but as of yet has not been observed in a stable form appropriate for clinical use.^47^ Astatine appears to be highly redox-active, and the highly ionizing nature of the emitted alpha radiation has been found to further contribute to the difficulty in deciphering astatine’s complex chemical nature.^48^ Alpha-particle-induced formation of peroxides has been shown to oxidize At^–^ to At^+^. At the same time, radiolysis of certain solvents, such as methanol, can result in the formation of reductive species like formaldehyde, which further complicates the overall redox picture.

**Figure 3:**
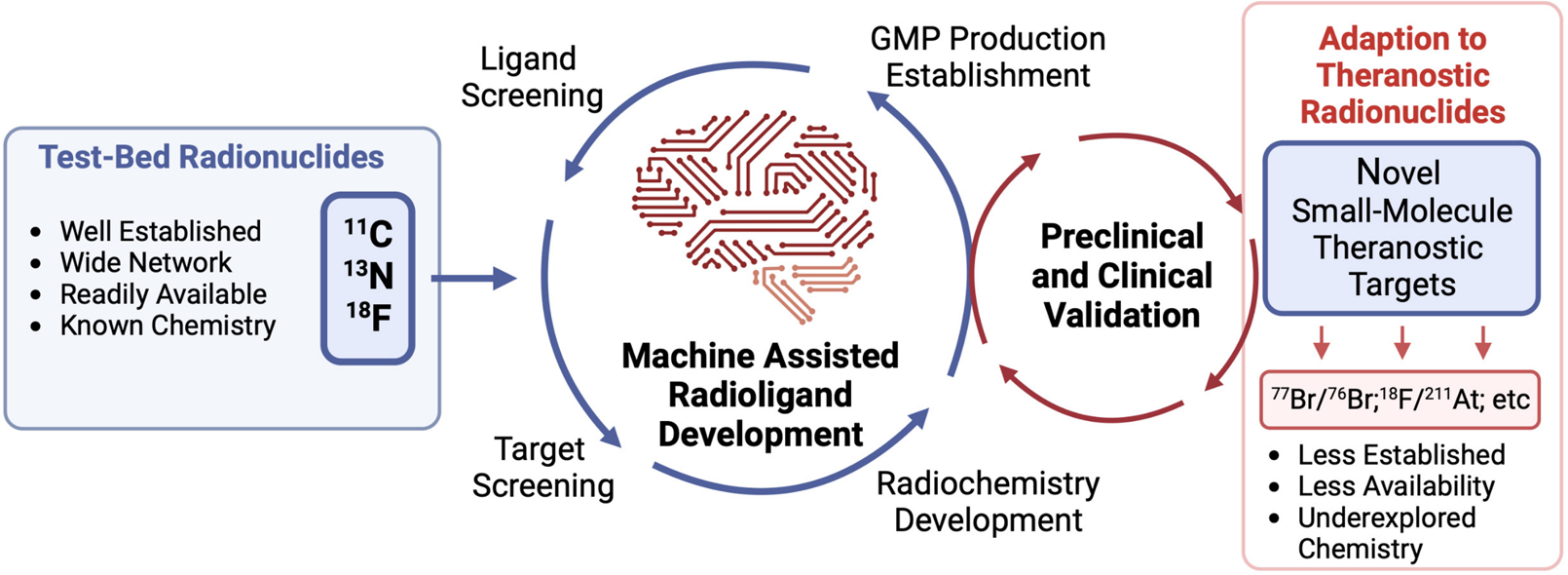
Well-established radionuclides ^11^C, ^13^N, and ^18^F and machine and data-science assisted radiopharmaceutical development will play a vital role in the discovery and development of novel theranostic targets and ligands. (Created using Biorender.com.)

Nevertheless, organic At-labeled compounds hold enormous potential as lipophilic α emitters capable of delivering high energy α-particles directly to sensitive intracellular components via small molecule targeting vectors.^49^ Moreover, due to the α-particle’s short path length, surrounding healthy tissue receives a much lower radiation dose. These advantages have driven a renewed interest in new astatination labeling methods. Traditionally, radioastatinated compounds have been prepared using electrophilic destannylation reactions from aryl tin precursors (Scheme 1A).^31^ However, as with bromine, recent studies have begun to adapt and improve upon modern metal-mediated radiolabeling methods for the astatination of small molecules, eliminating the need for the toxic traditional organotin precursors (Scheme 1B).^50^

While several ^211^At-labeled small molecules have been reported (Scheme 1C), C–At bonds have generally been found to be extremely labile in vivo, and solving the instability issues associated with astatinated radiopharmaceuticals is the most pressing area of astatine radiopharmaceutical research.^47^ There have also been several efforts to incorporate astatine into large biomolecules through the use of chelation approaches or through the conjugation of ^211^At into boron cage pendant groups; however, these approaches add significant bulk and charge to the final molecule.^51−53^ Moreover, the redox-sensitive nature of astatine further complicates its use in highly redox active in vivo environments, making the stability of At-radiopharmaceuticals a significant hurdle to their clinical application. Thus, the current focus of astatine-based radiopharmaceutical research is the development of stable astatine labeling strategies and prosthetic groups.

## 3 Coordination Chemistry of Short-Lived Radionuclides

**Scheme 2:**
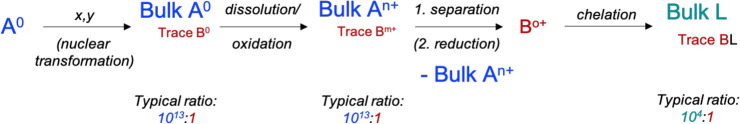
Typical Workflow for Production, Separation, Processing, and Chelation of Radionuclides That Form Coordinative Bonds

Aqueous coordination chemistry, dating back to the pioneering work of Alfred Werner, is perhaps the most extensively explored area of inorganic chemistry. However, there are significant limitations and challenges that are unique to radiocoordination chemistry and pose constraints on feasible chemical transformations. Specifically, the nature and speciation of the radiochemical precursor cannot be freely modified but typically hinges on the preceding separation from the target material. Concomitantly, the radionuclide may be accompanied by weakly coordinating buffer anions that determine solvent and pH conditions under which radiometalations must occur (Scheme 2).^54−56^ The tracer-level nature of radiopharmaceutical synthesis provides conditions where rate-laws are governed by the kinetic and thermodynamic behavior of the metal ion, and ligand concentration can delineate steady-state conditions.^57,58^ Reactivity profiles that produce near-quantitative yields in less than one radionuclide half-life are ideal. Below, we describe challenges that are in no way comprehensive, but rather representative of opportunities to develop new radiometal-coordination chemistry.

### 3.1 ^72/77^As: An Example of How a Knowledge Gap in Chemical Reactivity Results in Difficult-to-Control Biological Behavior

In addition to well-established and clinically utilized metalloid radioisotopes such as ^68^Ga, ^111^In, ^201^Tl, arsenic-72/77 has recently become of interest due to the ease of production of both isotopes and their value as a potential matched theranostic pair.^59^ Arsenic-72 (*β*^*+*^, *t*_1/2_ = 26 h) is suitable for PET, while ^77^As (*β*^*–*^, 227 keV, *t*_1/2_ = 38.8 h) represents the therapeutic match.^60,61^ In addition to direct production from the ^72^Ge (p, n) ^72^As reaction, Arsenic-72 can be produced from the decay of ^72^Se (*t*_1/2_ = 8.4 days) to form a ^72^Se/^72^As generator.^62^ Taken together, the ^72/77^As pair exhibits attractive properties from a nuclear medicine standpoint; however, the aqueous chemistry of As^(III)^ and As^(V)^ represents a radiosynthetic challenge,^63^ and the poor understanding of the pharmacokinetic behavior of both species in their mononuclear form adds further complexity to the interpretation of biological data.

#### 3.1.1 Chemical Bonding of Mononuclear As Species Is Understudied

Albeit a pnictogen, arsenic exhibits little overlap with the chemical reactivity of nitrogen and phosphorus; mononuclear arsenite and arsenate compounds are characterized by comparatively weak bonding interactions,^64^ amphoterism,^65^ and a propensity to form polynuclear or cluster systems.^66^ Due to the tracer nature of radiochemical applications, such constructs are not feasibly synthesized, and therefore mononuclear systems are of primary interest.

**Scheme 3:**
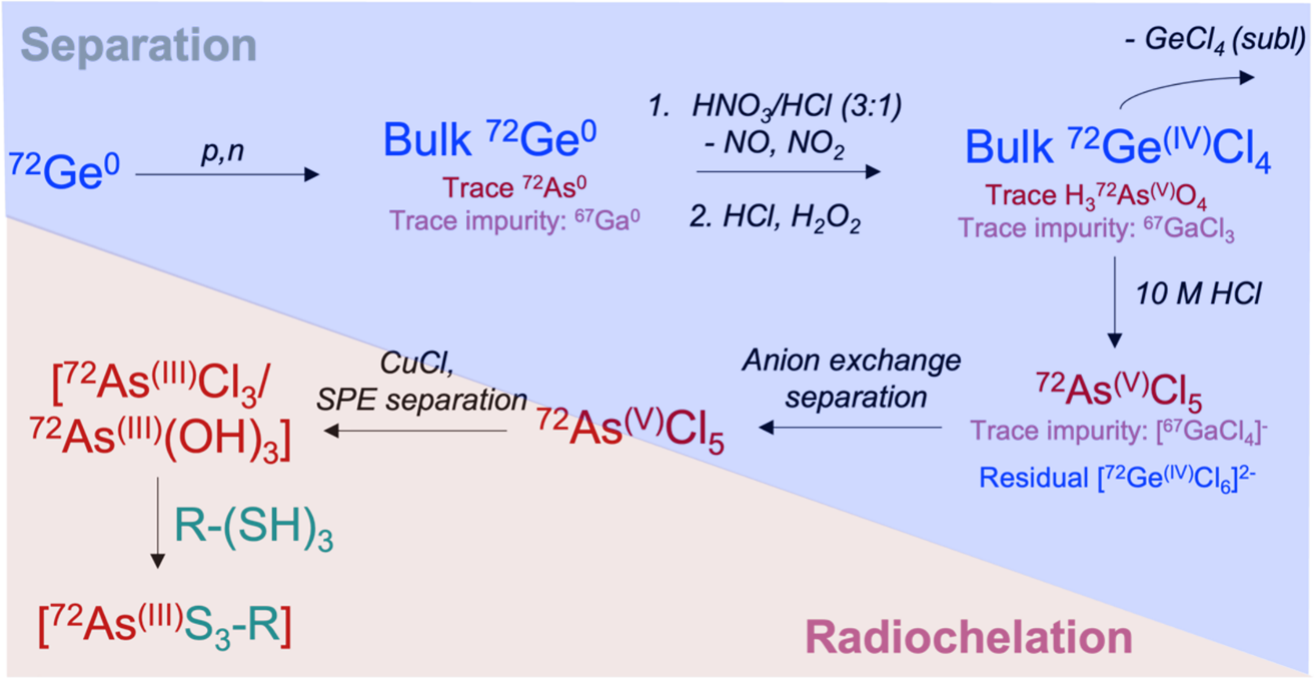
Production, Multistep Separation, and Chelation of ^72^As from a Proton-Irradiated, ^77^Ge Metallic Target

Radionuclide separations produce As^(V)^O_4_^3–^, which exhibits limited reactivity and means for functionalization; therefore, incorporation into more complex molecules is conducted by reduction to the more reactive As^(III)^ species (Scheme 3).^67^ The redox potential for the As^(III)^/As^(V)^ couple decreases from 0.3 V at pH 5 to nearly 0 V (versus Normal Hydrogen Electrode, NHE) at pH 7.4, which indicates that back-oxidation to arsenate represents a readily accessible decomposition pathway.^68^ As^(III)^ exhibits significant amphoteric behavior with a preference for soft donors like thiols or methenium.^69,70^ This pronounced thiophilicity results in binding to proteins with high cysteine content, which has previously been exploited to target and inhibit cysteine-rich oncogenic proteins by arsenic-containing anticancer drugs.^71,72^ Jurisson and co-workers have to date been most successful in developing chelator systems and radiolabeling approaches to stabilize and incorporate radioactive As^(III)^ using functionalized trithiol ligand systems.^67,73^ Preliminary in vivo experiments show hepatic localization, which can indicate enhanced lipophilicity, redox-mediated dechelation, or both.^74^

The thiophilicity of the As^(III)^ paired with weak binding interactions and poor shielding of the lone pair does not favor the formation of sufficiently strong and selective bonding interactions. Conclusively, controlling and stabilizing As^(III)^/As^(V)^ coordination complexes represent a veritable, fundamental coordination chemistry challenge. Only a handful of water-stable mononuclear As^(III)^/As^(V)^ complexes have been reported in the literature,^75,76^ with electrochemical investigation of ligand effects mostly lacking; this renders prediction of oxidation-mediated decomposition particularly difficult. Organometallic metalloid chemistry can provide inspiration on how to simultaneously stabilize the Lewis acidic and basic character of this ion—as demonstrated by O’Halloran and co-workers, transition-metal stabilized systems, where As^(III)^ is exploited for its amphoteric character, can be utilized.^77,78^

### 3.2 Chelation of Large, Low-Valent Ions with Poor Covalency: An Opportunity for Host–Guest Chemistry

The radionuclides ^201^Tl and ^223^Ra are part of a group of clinically employed radiopharmaceuticals in nuclear medicine that rely on the element’s intrinsic pharmacokinetic behavior and accumulation pattern for diagnostic and therapeutic efficacy. To date, their use is limited, but could be greatly expanded if methods are developed to incorporate these radionuclides into bifunctional, disease-targeting constructs.

#### 3.2.1 Challenges in Stabilizing Low-Valent, Large Cations

^201^Tl^(I)^ (γ, *t*_1/2_ = 3.03 days) is a SPECT agent employed to image and diagnose myocardial infarction. Localization relies on Tl^(I)^ mimicking Na^(I)^/K^(I)^ and utilization of the adenosine triphosphate (ATP) transport system in viable cells.^79^ The wide-ranging availability of ^201^Tl has recently resulted in the investigation of oxidation of Tl^(I)^ to Tl^(III)^ and subsequent chelation by amino-carboxylate chelators in close analogy to In^(III)^ chemistry.^80^ However, this approach has shown little success due to the difficulty in preventing back-reduction of Tl^(III)^ to Tl^(I)^ in aqueous media. Alternatively, a strategy that involves the selective capture and stabilization of the Tl^(I)^ ion employing its K^(I)^ character (ionic radii = 1.64 Å for Tl^(I)^, *r* = 1.55 Å for K^(I)^ and CN = 6)^81^ may be more promising, albeit remains challenging.^82^ Divergence of Tl^(I)^ from K^(I)^ chemistry is predominantly observed due to the relativistically contracted valence shell, the low electrical charge, and a stereoactive lone pair. Host–guest chemistry developed for K^(I)^ and transmembrane proteins evolved to bind K^(I)^ preferentially could further inform effective bifunctional chelator design.^83,84^

Challenges in incorporating ^223^Ra into bifunctional, targeted systems using selective and inert chelation have parallels to Tl-201. ^223^Ra is to date the only alpha emitting radioisotope that has reached FDA approval and finds wide-ranging application in clinical settings.^85−88^^223^Ra is produced by the decay of ^227^Th, which is the daughter of ^227^Ac (*t*_1/2_ = 27.1 years). ^227^Ac can be extracted from the waste of ^235^U mines and immobilized on an actinide chromatography resin to produce an ^227^Ac/^227^Th/^223^Ra generator.^89^ The decay of ^223^Ra produces 4 alpha particles and short-lived daughters (with half-lives less than 40 min) before reaching the stable daughter ^207^Pb, which allows for the deposition of a high dose of alpha particles in a short period of time within the target tissue. Due to the alpha recoil effect, the daughter nuclides would be ejected and effectively dissociated from the targeting moiety upon decay;^90^ however, displacement or recirculation of the daughter isotopes is minimized due to their short half-lives, further decreasing potential off-target effects. This contrasts with the decay chain of ^225^Ac, which includes ^209^Pb (half-life of 3.3 h), which may recirculate and potentially significantly damage healthy, nontarget tissues before further decay.^90^

^223^Ra is administered as a dichloride salt and relies upon the inherent chemical similarity of Ra^(II)^ and Ca^(II)^ to direct its accumulation to bone metastases caused by metastatic castration-resistant prostate cancer (mCRPC). The safety, production methods, and efficacy of ^223^Ra as a therapeutic have been established, and quantities to support clinical usage can be produced. Since its approval in 2013, over 18,000 patients have been treated with ^223^Ra, and the potential patient pool that could be treated would be significantly increased by expanding the use of ^223^Ra to treat other diseases.^91^ Furthermore, Ra^(II)^ qualifies as a nonvolatile, alkaline earth metal ion, and conceptually is amenable to aqueous coordination chemistry, potentially further simplifying the synthesis of radiopharmaceuticals once a sufficiently stable radiolabeling method can be established.

#### 3.2.2 A Need for a New Strategy to Stable Ra-Chelation

**Figure 4:**
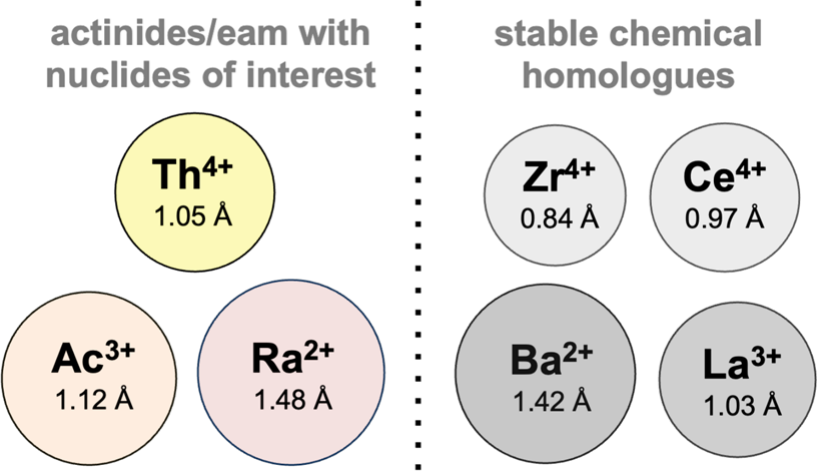
Ionic radius comparison of actinides and earth alkali metal ions of interest for nuclear medicine applications with cations of comparable ionic radius and chemical behavior.

Previous attempts to chelate radium have focused on small-molecule-based chelation strategies, specifically macrocyclic chelators such as macropa, crown ether functionalized calixarenes, and other polycyclic crown ethers.^91,92^ These systems were chosen based on their high affinity to Ba^(II)^ (ionic radius = 1.42 Å, CN= 8),^81,91,93^ a nonradioactive chemical surrogate of the Ra^(II)^ ion (ionic radius = 1.48 Å, CN = 8, Figure 4). However, none of the small molecule chelation strategies have led to the formation of Ra-complexes with appropriate in vivo stability. Rational chelator design remains difficult as key characteristics of radium, like the hydration state in solution and ideal coordination number, remain unknown, as no stable isotopes of Ra^(II)^ exist.

Recently, a unique calmodulin-like protein that binds lanthanides and actinides, lanmodulin (LanM), has been reported by Cotruvo and co-workers.^94^ Selective modification of a single amino acid within the metal ion binding site produced selective chelation of actinides over lanthanides, demonstrating that protein-based chelation methods are well suited for the development of metal-ion capture, separation, and stable chelation.^95^ Due to the similarity of Ca^(II)^ with Ba^(II)^ and Ra^(II)^, modification of the native calmodulin protein as a basis for a mutant library to develop Ra^(II)^ selective proteins may be explored. Residues may be mutated within the calcium-binding site to produce increased affinity and high selectivity for Ra^(II)^ over Ca^(II)^; similarly, a Mn^(II)^ selective LanM variant has already been reported.^96^

### 3.3 Chemistry of metal ions without stable isotopes: actinides

Other isotopes with a potential for clinically translatable alpha therapies are ^225^Ac, ^227^Th, and ^212^Pb. ^225^Ac, ^227^Th, and ^212^Pb require nonstandard production and suffer from supply chain issues that severely limit access to these isotopes for a large clinical patient population. For instance, primary production methods of ^225^Ac rely on the decay of ^229^Th (half-life 7340 years), producing only 1.7 Ci per year, which is not enough to support large scale clinical applications.^90^ Additional methods, such as the spallation of ^232^Th, which produces the long-lived ^227^Ac (*t*_1/2_ = 21.8 years), are currently under consideration but will require scale-up of production to broaden patient access to alpha therapies.^97^ Interest in alpha therapy applications has spurred recent developments in the aqueous coordination chemistry of both actinides that have only one relevant oxidation state under aqueous conditions (Ac^(III)^ and Th^(IV)^). Model systems with lighter congeners may be used but may have limited predictive nature (Figure 4); thus, work with the longest-lived isotope is most constructive. While this is challenging for Ac^(III)^, Th^(IV)^ possesses various long-lived isotopes that can be considered observationally stable, such as ^232^Th (*t*_1/2_ = 1.405 × 10^10^ years).

#### 3.3.1 Actinium Is the Largest Trivalent Metal Ion and Poses Unique Challenges

The largest lanthanide La^(III)^ is used as a stable congener for Ac^(III)^, which has proven mostly reliable, yet with the caveat that the coordination number of the corresponding Ac^(III)^ complex may be expanded by coordination of inner-sphere water molecules that are not present for the La^(III)^ analog (Figure 4).^98^ Study of the Ac^(III)^ aqua ion has indicated that the Ac(H_2_O)_*y*_^3+^ complex likely exhibits *y* = 10–11 with Ac–O bond lengths of 2.544 to 2.845 Å as determined by EXAFS analysis and an ionic radius of 1.12 Å for this species,^99,100^ and an estimated 1.065 Å for coordination number (CN) 6.^101^ This contrasts established values of La^(III)^ significantly, which have been reported as *y* = 9, with La–O bond lengths of 2.54 Å, and an ionic radius of 1.03 Å for CN = 6. Electrostatic interactions and steric constraints drive chelation for Ac^(III)^ in the absence of stabilization by the ligand field or coordinative bond covalency. Most successful, low-temperature chelation strategies rely on larger cavity crown-type macrocycles achieving coordinative saturation by incorporation of additional intermediate hardness mono- and bidentate donors such as acetate and picolinate.^98,102,103^ Additional approaches by smaller-cavity caged bispidines (Comba)^104^ and flexible acyclic polydentate chelators (Orvig)^105^ have indicated some success under these conditions as well. However, 8-coordinate chelators such as DOTA and corresponding phosphonate functionalized variants (DOTP) can also be used to form kinetically inert, in vivo compatible coordination complexes, albeit they require elevated temperatures to form in-cage complexes with all f-elements.^106^ Macromolecular, protein-based approaches to Ac^(III)^ chelation may also provide a suitable alternative that has not extensively been characterized to date. For instance the ^228^Ac^(III)^-lanmodulin complex forms the strongest actinide^(III)^-protein complex (subpicomolar *K*_d_) to date.^107−109^ The protein’s favorable properties allowed applications to separation from of >10^+10^ equivalents of divalent and tetravalent competing ions. While radiolabeled lanmodulin complexes have not shown sufficient inertness in vivo, further stabilizing modifications to the metal-bound, folded protein may afford structures with improved in vivo performance.

#### 3.3.2 Thorium Is the Only Tetravalent f-Element without Relevant Redox Chemistry in Water

Thorium is the most abundant radioactive element on earth, yet it exhibits reactivity drastically different from other f-elements and other elements with a stable tetravalent oxidation state. Bonding in Th^(IV)^ is predominantly ionic with considerable d-orbital involvement and characterized by a preference for hard donors due to its high Lewis acidity.^110,111^ Th^(IV)^ has an ionic radius of 1.05 Å (CN = 8), making it the largest observationally stable tetravalent metal ion.^81^ Stable elements with relevant tetravalent oxidation states and comparable ionic radius are Ce^(IV)^ (0.97 Å for CN = 8) or Hf^(IV)^ (0.83 Å for CN = 8) and Zr^(IV)^ (0.84 Å for CN = 8) but can differ significantly in their coordinative and electrochemical behavior. The comparatively large ionic radius and more diffuse charge distribution of Th^(IV)^ result in a preference for 8-coordinate, Lewis basic donor systems and a diminished tendency for hydrolytic behavior when compared to tetravalent transition metal ions with a smaller ionic radius such as Hf^(IV)^ or Zr^(IV)^. In recent years, polydentate, Lewis basic ligand systems such as 3,4,3-LI(CAM) or 3,4,3-LI(1,2-HOPO), and functionalized Me-3,2-HOPO-based systems have shown promising performance for the chelation of Th^(IV)^.^112−114^ Pilot treatment efficacy studies of corresponding antibody-conjugates in mice show promising results, indicating that the development of tailored chelation approaches specifically to Th^(IV)^ is warranted.^115^

### 3.4 From Inert to Dynamic: Radiochemistry of Redox-Active Transition Metals

**Figure 5:**
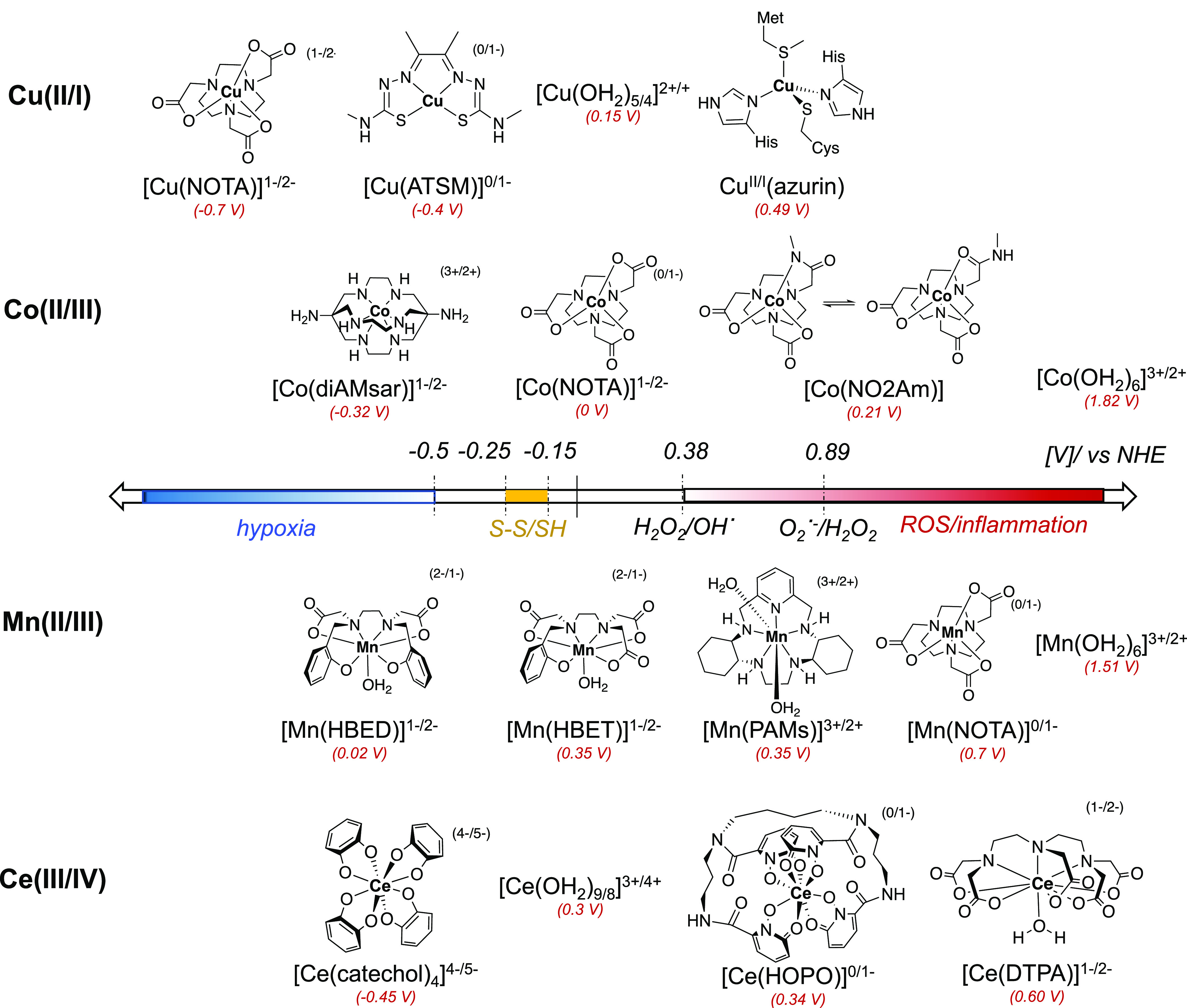
Electrochemical redox potential of commonly employed and well-established coordinative environments of radiometal ions of interest, shown in the context of biologically relevant oxidants and reductants. Values are provided vs normal hydrogen electrode (NHE) and in all cases based on values measured in >50% aqueous media.

In contrast to heavy elements of interest vide supra, first row transition metal ions provide ample opportunity for extensive spectroscopic characterization on the macroscopic scale and a large body of literature to draw from with respect to well-characterized chelation approaches. Their prevalence in biology also provides unprecedented access to the study of metallo-homeostasis in healthy and diseased states. With respect to availability, copper PET radionuclides ^62^Cu (*β*^*+*^, *t*_1/2_ = 0.16 h), ^64^Cu (*β*^*+*^, *t*_1/2_ = 12.7 h), and the therapeutic nuclide ^67^Cu (*β*^*–*^, *t*_1/2_ = 61.8 h) are most established and have been incorporated into clinical radiopharmaceuticals.^116−118^ More recently, the production of radionuclides of manganese and cobalt, ^52^ Mn (β^+^, *t*_1/2_ = 134.4 h),^119−121^ as well as ^55^Co (β^+^, *t*_1/2_ = 17.5 h), and ^58m^Co (*t*_1/2_ = 9.1 h, IC, Meitner-Auger electron) using low energy cyclotron production pathways have been established.^122,123^ All three elements have widely accessible redox chemistry under physiological conditions, providing both a challenge and opportunity to achieve selective deposition of the radioactive payload in target tissues.^124^ This has been recognized and subsequently extensively demonstrated with Cu^(II)^ complexes with comparatively low reduction potentials, such as Cu(ATSM) (*E*_1/2_ = −0.59 V, vs NHE) and Cu(PTSM) (*E*_1/2_ = −0.51 V vs NHE), which have both been translated clinically.^125,126^ Their mechanism of action relies on the dechelation-mediated accumulation of the Cu^(I)^ species, following internalization and subsequent reduction of the Cu^(II)^-species in hypoxic environments (Figure 5).^127^

While this mechanism has been validated, challenges are posed by the endogenous trafficking of the liberated copper ion. Possible redox-responsive agents may be desirable, where reduction does not result in dechelation of the radioactive ion. Indeed, the redox-chemistry of water-compatible, mononuclear Mn^(II)^/Mn^(III)^ and Co^(II)^/Co^(III)^ coordination complexes are rather well explored in the context of paramagnetic chemical exchange saturation transfer (CEST) and magnetic resonance imaging (MRI) contract agents. Redox potentials of Mn and Co species range 0.1–0.6 V and −0.2–0.8 V (vs NHE), respectively; this means that redox-responsive species must be formed and administered in the (III)-oxidation state and respond to reducing environments by controlled reduction to the (II) species.^128−130^ The validation of such systems has been shown for the above-mentioned applications using stable nuclides but is complicated in the case of short-lived radionuclides by the production and separation chemistry, which produces the ready-to-chelate ion in the (II)-oxidation state. Conclusively, the corresponding complex must be formed and subsequently oxidized and stabilized in the higher-valent state prior to administration. In general, a half-cell potential of −0.5 V (vs NHE) is considered the threshold for an appropriately responsive agent in hypoxic environments, whereas reactive oxygen species and thiols range from 0.3 V and −0.25 to −0.15 V, respectively (vs NHE).^131^ Nonequilibrium conditions, caused by oxidants and reductants being present at 2–50-fold excess, apply to tracer chemistry and can further modulate the lifetime of transiently stable species in ways that cannot be replicated under macroscopic conditions. Consequently, harnessing the unique concentration differences of noncarrier added, tracer conditions of redox active species provide potential applications as redox-activatable radiotheranostics.

Another application of redox-potential plasticity under physiological conditions is the stabilization of different oxidation states of diagnostic nuclides to mimic the pharmacokinetic behavior and valency of a therapeutic nuclide without imageable emissions. This has been shown by modulation of the Ce^(III)/(IV)^ couple with the ^134^Ce isotope (IC< *t*_1/2_ = 75.8 h), which decays to short-lived ^134^La (β^+^, *t*_1/2_ = 6.7 min).^132^ Abergel, Kozimor, and co-workers demonstrated that the oxidation state of the cerium species was determined by the chelator system used, producing Ce^(III)^ using softer donors such as DTPA and Ce^(IV)^ with 3,4,3-LI(1,2-HOPO) under physiological labeling conditions.^133,134^ Subsequently, Ce^(III)^ can be used as an imaging surrogate to Ac^(III)^, whereas Ce^(IV)^ recapitulates the chemical behavior of Th^(IV)^, acting as the imaging partner to either therapeutic nuclide. In addition to harnessing redox-plasticity to form theranostic pairs with Ac^(III)^ and Th^(IV)^, it may also be possible to exploit the electrochemical behavior of the Ce^(III)/(IV)^ couple in vivo.

## 4 Conclusions

The resurgence of radiochemistry in the wake of the FDA approval of theranostics and vastly improved access to cyclotron use for radionuclide production provides wide-ranging opportunities for the discovery and application of new chemistry at the tracer level. Despite the increasing diversity of chemical elements available for experimentation, the eventual clinical translation is not guaranteed and must remain decoupled from the research and development process. Radionuclide production, separation, and radiochemical synthesis require a careful, analytical, experimental approach at the interface of medical physics, materials science, and organic and inorganic chemistry. Elements without long-lived, stable isotopes pose a special challenge, where clinical translation will be preceded by the elucidation of so far unknown, fundamental chemical reactivity.

## Abbreviations

DOTA: 2,2′,2″,2‴-(1,4,7,10-tetraazacyclododecane-1,4,7,10-tetrayl)tetraacetic acid
DOTP: 1,4,7,10-tetraazacyclododecane-1,4,7,10-tetra(methylene phosphonic acid)
EXAFS: extended X-ray absorption fine structure
FDA: Food and Drug Administration
FDG: [^18^F]fluorodeoxyglucose
MIBG: meta-iodobenzylguanidine
PET: positron emission tomography
PSMA: prostate specific membrane antigen
SPECT: single photon emission computed tomography
SSTR2: somatostatin receptor 2
